# The psychometric properties of positive and negative beliefs about the rumination scale in Chinese undergraduates

**DOI:** 10.1186/s40359-023-01111-8

**Published:** 2023-04-11

**Authors:** Honggui Zhou, Hong Liu, Xiaohong Ma, Yunlong Deng

**Affiliations:** 1 Psychosomatic Health Research Institute, The Third Xiangya Hospital, Central South University, Changsha, China; 2 Faculty of Psychology, Beijing Normal University, Beijing, China; 3 Education Center for Mental Health, Central South University, Changsha, China

**Keywords:** Depressive rumination, Metacognition, PBRS, NBRS, Psychometric property

## Abstract

**Background:**

Rumination, a transdiagnostic factor in different psychopathological conditions, is believed to be activated and sustained by dysfunctional metacognition. The Positive Beliefs about Rumination Scale (PBRS) and the Negative Beliefs about Rumination Scale (NBRS) have been used to measure the metacognitive beliefs of rumination and have been investigated in many cultural contexts. However, it remains unclear whether these scales can work as well for the Chinese population. Therefore, this study aimed to explore the psychometric properties of the Chinese versions of these scales and to test the metacognitive model of rumination for students with different levels of depression.

**Methods:**

The PBRS and NBRS were forward-backward translated into Mandarin. In total 1,025 college students were recruited to complete a battery of web-based questionnaires. Exploratory factor analysis, confirmatory factor analysis, and correlation analysis were used to test the structure, validity, and reliability of the two scales, as well as their item correlations with rumination.

**Results:**

A new two-factor structure of the PBRS (rather than the original one-factor model) and a new three-factor structure of the NBRS (rather than the original two-factor model) were extracted. The goodness-of-fit indices of these two factor models showed they had a good to very good fit with the data. The internal consistency and construct validity of PBRS and NBRS were also affirmed.

**Conclusion:**

The Chinese versions of the PBRS and the NBRS were generally shown to be reliable and valid, but their newly extracted structures fit the Chinese college students better than their original structures. These new models of PBRS and NBRS are of value to be further explored in Chinese population.

## Introduction

Rumination, defined as self-focused, persistent, depressive thinking, occurs as a response to initial negative thoughts in both normal individuals and clinical patients [1]. Studies have revealed the predictive effects of rumination on the onset of depression, that rumination interacts with negative cognitive styles to predict the duration of depressive symptoms [2], and that rumination is a possible transdiagnostic mediator of vulnerability and outcome in different psychopathological conditions [3, 4].

Why do people use this maladaptive emotion regulation strategy in response to their negative affect? According to the Self-Regulation Executive Function (S-REF) model [5], it might be activated and guided by metacognitive beliefs [6], which are stable beliefs people have about their own cognitive systems. Metacognitive beliefs are usually classified into two types [7]: positive beliefs that refer to the utility of rumination and negative beliefs that refer to the danger, uncontrollability, and terrible consequences of rumination. Different types of metacognitive beliefs can influence the control, monitoring, and appraisal of cognition in different ways. The occurrence of negative experiences activates positive metacognitive beliefs about the usefulness of rumination (threat monitoring), leading people to use rumination to overcome a negative situation and help understand a problem or solve it. Negative metacognitive beliefs, on the other hand, lead to the follow-up activation of perseverative thinking and deteriorative depressive symptomatology [8].

Moreover, many studies have provided supportive evidence for the effects of metacognitive beliefs on rumination and depression. Previously depressed patients have reported higher metacognitive awareness than currently depressed individuals to avoid depressive episodes [9]. Positive beliefs about rumination can predict engagement in rumination and postpartum depression through negative beliefs about rumination [10]. In addition, regressive analysis has shown that the relationship between positive beliefs and depressive symptoms is fully mediated by rumination, while the relationship between negative beliefs was partially mediated by rumination [11].

Furthermore, this metacognition theory also proposes that dysfunctional metacognition is associated with the presence of transdiagnostic psychological distress [12, 13]. It is also accompanied by activities related to cognitive attentional syndrome (CAS), which comprises perseverative thinking (e.g., rumination and worry), predominantly strategic attentional bias (e.g., focusing on negative feelings, thoughts, and threats), and other unhelpful coping strategies (e.g., avoidance of activities) that are inadvertently intensified and that prolong emotional responses [14]. Research has shown that metacognitive beliefs can activate and sustain unhelpful coping styles and become a vulnerability factor for emotional and psychological problems [15], such as affective disorders [6], procrastination [16], obsessive–compulsive disorder (OCD) [17], addictive behaviors [18], and psychosis [19].

Metacognitive therapy (MCT), a psychotherapy based on the S-REF model and focused on metacognitive processes and metacognitive beliefs [20], have been shown to reduce dysfunctional metacognition, rumination [21], and depressive symptoms [22]. Some studies have even suggested that the effect of MCT in treating depression is superior compared to cognitive behavioral therapy [23]. These positive effects of MCT were found to be sustained in follow-up assessments for months [24, 25] and even over years [26, 27].

Valid instruments to assess metacognition are needed to verify these mechanisms and to propose effective interventions for individuals struggling with mental disorders. The Meta-Cognitions Questionnaire (MCQ) was designed specifically to measure metacognitive beliefs about worry [28] and to measure metacognitive beliefs in studies of depression [29, 30]. Although worry and rumination are related and have a relationship with both depression and anxiety, factor analysis [31] has shown that worry and rumination are distinct cognitive processes, and the factors (e.g., dwelling on the negative) taped more maladaptive component of rumination compared to worry. Therefore, it is necessary to develop and revise a valid instrument to measure metacognitive beliefs about rumination.

The metacognitive measurements used to target depressive rumination were the Positive Beliefs about Rumination Scale (PBRS) [1] and the Negative Beliefs about Rumination Scale (NBRS), whose items were both derived from reports by patients with major depressive disorder [32]. Following a series of studies by Papageorgiou and Wells in non-clinical and clinical samples, the original PBRS was confirmed with a one-factor structure, and the NBRS comprised two different subscales: negative beliefs about the uncontrollability and harmfulness of rumination (NBRS1) and negative beliefs about the social and interpersonal consequences of rumination (NBRS2) [7]. All the initial PBRS, NBRS1, and NBRS2 had good internal consistency (0.89, 0.80, 0.83 respectively), and good psychometric properties of validity [33]. In Turkish samples, the psychometric properties and original structure of the PBRS and NBRS have been confirmed [34]. The Croatian version of PBRS and subscales of NBRS also reported good internal consistency [7]. In terms of Eastern cultures, only the Japanese version of the PBRS has been investigated [35].

The majority of the above studies were from Western countries. The influential differences between Western and Chinese culture, however, have not been neglected. Studies have suggested that Chinese patients with depressive disorder tend to emphasize their somatic symptoms, such as pain, dizziness, or fatigue, rather than their psychological status, whereas Western depressed patients tend to report their subjective feelings, including feeling sad, upset, and frustrated, rather than their physical problems [36–38]. Unlike Western culture, which emphasizes the personal experience and interpersonal communication of emotion [36, 38, 39], Chinese culture encourages individuals to show self-restraint rather than express feelings, which may impact an individual’s behavior and attitudes toward mental health issues [40]. Considering the differences and the influence of social and historical environments on people’s psychological capabilities, whether the original structures of PBRS and NBRS remain stable across different cultures needs to be investigated.

Considering the exceeding high prevalence of depression among Chinse college students [41], and the predictive effect of rumination on depressive symptoms in them [42], it is imperative to pay more attention to the development of appropriate measurements and interventions to take care their mental health. In the current study, therefore, we aimed to explore the psychometric properties of the Chinese versions of the PBRS and the NBRS in university students to verify these scales’ cross-cultural applications.

## Methods

Before carrying out the translation, agreement was obtained from the first author of these two scales [1]. The original versions of the PBRS and the NBRS were forward-translated by a group of psychology doctoral candidates and professors who didn’t know the construct. The Chinese versions of the PBRS and the NBRS were back-translated by another psychological doctoral candidate who was studying in an English-speaking country and blinded to the original questionnaires.

### Participants and procedure

We used convenience sampling (i.e., online advertisements) to collect data from a university in Changsha, China. Undergraduates who voluntarily participated in our study were asked to complete a battery of web-based questionnaires. All participants were provided with informed consent and instructed to answer truthfully. When considering the participants’ response time, researchers believed that it was unlikely for participants to respond to survey items at a rate faster than 2 s per item [43]. In our study, the online survey consisted of consent, the introduction of each questionnaire, 108 items in total, and a brief acknowledgement. Participants who finished the whole battery within five minutes or gave invalid answers (e.g., responded the same way on all items) [44] [46]were excluded. Ultimately, 1,025 participants (498 males) completed the questionnaires without missing data. The participants’ mean age was 19.59 ± 1.39 (mean ± SD) years. Three weeks later, 129 participants (54 males, mean age = 19.08 ± 1.23) completed the Chinese versions of the PBRS and the NBRS to evaluate their test-retest reliability. No sex ratio difference was found between the two samples (Pearson ${\chi }^{2}\left(1\right)$ = 2.077, *p* = 0.15). Participants’ age ranges in the first and re-test samples were 16 to 25 and 17 to 22, respectively. 89.85% of participants were aged from 18 to 21 in the first sample, while the percentage of participants who were in the same range in the re-test sample was 95.35%. No age difference was found between the two samples (${\chi }^{2}\left(3\right)$ = 6.711, *p* = 0.082).

### Instruments

The PBRS [1] is a 9-item scale assessing positive metacognitive beliefs about rumination (e.g., “Rumination about the past helps me to avoid mistakes and failures in the future”). The NBRS [32] is a 13-item questionnaire assessing negative metacognitive beliefs about rumination. The original version of the NBRS comprised two subscales: beliefs about the uncontrollability and harmfulness of rumination (oNBRS1, e.g., “Ruminating is uncontrollable”), and beliefs about the interpersonal and social consequences of rumination (oNBRS2, e.g., “Ruminating causes me to be rejected by others”). Participants were asked to indicate the extent to which they agree with each of the items using a 4-point Likert-type scale ranging from 1 (do not agree) to 4 (agree very much). In previous studies, the total score of PBRS and NBRS were recommended to measure individual’s positive or negative beliefs about rumination.

The Center for Epidemiologic Studies Depression scale (CES-D) [45] is a 22-item self-report questionnaire widely used in assessing the severity of depressive symptoms. Items are rated on a 4-point Likert-type scale ranging from 0 to 3. The Chinese CES-D has shown excellent reliability and validity (Cronbach’s α = 0.913 in this study) [46].

The Ruminative Response Scale (RRS) [47] is a 22-item scale measuring individuals’ different tendency to respond to depressed mood. Items are rated on a 4-point Likert-type scale ranging from 1 to 4. The Chinese version of the RRS is a reliable measure of rumination that has shown excellent internal consistency (Cronbach’s α = 0.926 in this study) [48].The Penn State Worry Questionnaire (PSWQ) [49] is a 16-item scale assessing individuals’ vulnerability to engage in generalized, excessive, and uncontrollable worry. Items are rated on a 5-point Likert-type scale ranging from 1 to 5. The Chinese version of the PSWQ was shown to be valid and has good reliability (Cronbach’s α = 0.899 in this study) [50].

The Meta-Cognitions Questionnaire-30 (MCQ-30) [51] is a short form of the original MCQ that consists of 65 items on five subscales. Subscale 2 (MCQ2) concerns positive beliefs about worry (e.g., “Worry helps me to solve problems”). Subscale 4 (MCQ4) concerns negative beliefs about the uncontrollability and danger of worry (e.g., “I cannot ignore my worrying thoughts”). These two subscales of the Chinese version of MCQ-30 have shown acceptable to good reliability and validity (Cronbach’s α = 0.882 for MCQ2 and Cronbach’s α = 0.799 for MCQ4 in this study) [52].

### Data analysis

Statistical analyses were carried out using the Statistical Package for the Social Sciences (SPSS version 23) and Mplus software (Mac version 7.4) [53]. The total sample was randomly divided in two samples to evaluate the factorial structure of the PBRS and NBRS: an exploratory sample (n = 513) and a confirmatory sample (n = 512). The former sample was used to carry out an exploratory factor analysis (EFA) to identify the factor structure of the scales. The latter sample was adopted to perform confirmatory factor analysis (CFA), using the Weighted Least Squares Median-adjusted (WLSM) method because scales were generally not considered as continuous ones if their items were rated on a 4-point Likert-type scale [54].

After exploring the structures of PBRS and NBRS, a series of correlation analysis were conducted to assess the validity of the two scales [55]. For the concurrent validity, correlation analysis was conducted between the two scales and the subscales of MCQ (MCQ2 and MCQ4). For the convergent validity, correlation analysis was performed between the two scales and CES-D and RRS. For the divergent validity, the correlation coefficients between the two scales and RRS were compared to the correlation coefficients between the two scales and the PSWQ [33].

Finally, the Cronbach’s alpha coefficient was used to test the internal consistency. The intra-class correlation coefficient (ICC) was used to evaluate the test–retest reliability of these two scales. According to previous studies, the acceptable values of alpha were ranging from 0.70 ~ 0.95 [56], and 0.70 was recommended as a minimum standard for reliability [57]. In addition, independent *t*-tests were also performed to examine the potential gender differences.

## Results

### Item analysis

Item-total statistics were used to test the homogeneity of these two scales. The results showed that all the corrected item-total correlations ranged from 0.67 to 0.83 for the PBRS and from 0.42 to 0.70 for the NBRS.

### Construct validity

The EFA was performed on scores from a randomly selected subsample (n = 513). The adequacy of Kaiser–Meyer–Olkin (KMO) (0.900 for the PBRS and 0.868 for the NBRS) and significance of Bartlett’s test of sphericity (χ^2^ = 2383.357 for the PBRS, χ^2^ = 2184.768 for the NBRS, both *p* < 0.001) verified the appropriateness of the sample for factor analysis [58]. As seen in Table 1, for the PBRS, two factors were extracted, accounting for 66.797% of the total variance (PBRS1 = 11.507%, PBRS2 = 55.291%). For the NBRS, three factors were extracted, accounting for 56.504% of the total variance (NBRS1 = 37.147%, NBRS2 = 7.914%, NBRS3 = 11.442%). Factor loadings were all greater than the 0.4 cutoff variance. The results showed that the loading of each item on its factor ranged from acceptable to good [59].

**Table 1:** Factor loading for PBRS and NBRS items in pattern matrix (n = 512)

Item	PBRS1	PBRS2	Item	NBRS1	NBRS2	NBRS3
p1	**0.972**	-0.186	n1	**0.798**	0.106	-0.251
p2	**0.846**	-0.030	n2	**0.820**	0.034	-0.244
p3	**0.835**	0.057	n3	**0.789**	-0.052	0.037
p4	0.385	0.386	n4	**0.627**	-0.069	0.266
p5	-0.196	**0.917**	n5	**0.529**	-0.123	0.342
p6	0.373	**0.544**	n6	0.099	**0.691**	0.041
p7	0.174	**0.693**	n7	0.307	0.148	0.250
p8	0.272	**0.606**	n8	0.228	0.033	**0.631**
p9	-0.154	**0.912**	n9	0.199	**0.456**	0.163
			n10	-0.301	0.077	**0.872**
			n11	-0.106	**0.867**	-0.037
			n12	-0.139	-0.058	**0.897**
			n13	0.215	0.027	**0.585**

Note: Prefixes of the items (letter “p” or “n”) indicates which scale the items belong to (p for PBRS, n for NBRS). Bold data indicates which factor the items belong to

According to the results of the EFA and the content of the factors, two factors of the PBRS were labeled as follows: PBRS1 (3 items): Understanding feelings and causes; PBRS2 (5 items): Problem discovering and preventing. Three factors of the NBRS were labeled as follows: NBRS1 (5 items): Consequences of rumination; NBRS2 (3 items): Uncontrollability of rumination; NBRS3 (4 items): Self-appraisal with rumination. The factor loading of item 4 of the PBRS and item 7 of the NBRS were found to be below 0.4; consequently, they were deleted in this sample.

The CFA was performed with data from the remaining sample (n = 512), both for the new models resulted from the EFA and the original models referred to the original scales. When testing the two-factor model of the PBRS and the three-factor model of the NBRS, an examination of the modification indices revealed that two items (items 5 and 9) in the PBRS and two items (items 4 and 5) in the NBRS had significant residual correlations. Therefore, modified models of these two scales were tested after removing item 4 from the PBRS and item 7 from the NBRS, allowing items 5 and 9 of the PBRS and items 4 and 5 of the NBRS to have residual correlation. As the results in Table 2 shown, though the original one-factor model of the PBRS and the original two-factor model of the NBRS were acceptable, the new two-factor model of PBRS and three-factor of NBRS showed significant improvement and had a good to very good fit with the data.

**Table 2:** Goodness-of-Fit indices of the PBRS and NBRS factor models (n = 513)

Model	χ^2^ (*df*)	CFI	TLI	RMSEA	90% CI RMSEA
PBRS (original one-factor model)	413 (27)	0.956	0.943	0.167	0.153–0.182
PBRS (two-factor model)	211 (19)	0.976	0.965	0.141	0.124–0.158
**PBRS (modified two-factor model)**	**53 (18)**	**0.996**	**0.993**	**0.062**	**0.043–0.081**
NBRS (original two-factor model)	291 (64)	0.951	0.940	0.083	0.074–0.093
NBRS (three-factor model)	206 (51)	0.964	0.953	0.077	0.066–0.088
**NBRS (modified three-factor model)**	**153 (50)**	**0.976**	**0.968**	**0.064**	**0.052–0.075**

Note: PBRS = Positive Beliefs about Rumination scale, NBRS = Negative Beliefs about Rumination Scale

### Concurrent, convergent, and divergent validity

Pearson correlations between the modified subscales of PBRS and NBRS are shown in Table 3, as well as their original version. A significant large positive relationship was found between the two subscales of the PBRS and the three subscales of the NBRS. A significant minor to moderate positive relationship was found between the subscales of the PBRS and the NBRS. The correlation between mPBRS1 and mNBRS, mNBRS1, mNBRS2, and mNBRS3 was significantly higher than the correlation between mPBRS2 and these NBRS subscales (*z* = 6.063, 5.113, 4.435, and 4.997; all *p* < 0.001).

**Table 3:** Correlation matrix of modified PBRS and NBRS, original PBRS and NBRS, and self-report scales (n = 1025)

	mPBRS	mPBRS1	mPBRS2	mNBRS	mNBRS1	mNBRS2	mNBRS3
mPBRS1	0.874^**^	-					
mPBRS2	0.947^**^	0.671^**^	-				
mNBRS	0.328^**^	0.388^**^	0.244^**^	-			
mNBRS1	0.267^**^	0.320^**^	0.196^**^	0.903^**^	-		
mNBRS2	0.116^**^	0.173^**^	0.062^*^	0.736^**^	0.556^**^	-	
mNBRS3	0.390^**^	0.428^**^	0.312^**^	0.779^**^	0.519^**^	0.397^**^	-
CESD	0.211^**^	0.283^**^	0.135^**^	0.453^**^	0.369^**^	0.426^**^	0.313^**^
MCQ2	0.366^**^	0.245^**^	0.396^**^	0.064^*^	0.005	0.172^**^	-0.015
MCQ4	0.321^**^	0.359^**^	0.252^**^	0.575^**^	0.466^**^	0.549^**^	0.390^**^
RRS	0.485^**^	0.492^**^	0.414^**^	0.507^**^	0.423^**^	0.488^**^	0.315^**^
PSWQ	0.314^**^	0.341^**^	0.253^**^	0.435^**^	0.318^**^	0.479^**^	0.277^**^
*z*-test	6.224^**^	5.555^**^	5.647^**^	2.753^**^	3.752^**^	0.348	1.309

Note: mPBRS = modified Positive Beliefs about Rumination Scale, mPBRS1 = Subscale 1 of modified PBRS (Understanding feelings and causes), mPBRS2 = Subscale 2 of modified PBRS (Problem discovering and preventing), mNBRS = modified Negative Beliefs about Rumination Scale, mNBRS 1 = Subscale 1 of modified NBRS (Consequences of rumination), mNBRS2 = Subscale 2 of modified NBRS (Uncontrollability of rumination), mNBRS3 = Subscale 3 of modified NBRS (Self-appraisal with rumination), CES-D = the Center for Epidemiologic Studies Depression scale, MCQ2 = positive beliefs about worry, MCQ4 = negative beliefs about thoughts concerning uncontrollable and danger, RRS = Ruminative Response Scale, PSWQ = Penn State Worry Questionnaire. ^**^ significant at the 0.01 level. ^*^ significant at the 0.05 level

Evidence supporting the concurrent validity of the modified PBRS (mPBRS) was provided by a significantly higher correlation with MCQ2 than with MCQ4 (*z* = 2.212, *p* < 0.05), in which MCQ2 was used to measure the positive beliefs about worry and MCQ4 was used to measure negative beliefs about the uncontrollability and danger of worry. The significant positive correlations between mPBRS and CES-D/RRS, which measured the severity of depressive symptoms or rumination, supported for the convergent validity of the mPBRS. The divergent validity of mPBRS was also affirmed by significantly higher correlations with the RRS than with the PSWQ (*z*-test in Table 3), which specifically measures proneness to anxious worry. Specifically, for the two subscales of mPBRS, the correlation between PBRS2 and MCQ2 was significantly higher than the correlation between PBRS2 and MCQ4 (*z* = 2.702, *p* < 0.01), while the correlation between PBRS1 and MCQ2 was significantly lower than the correlation between PBRS1 and MCQ4 (*z* = -3.847, *p* < 0.01). Furthermore, the correlation between PBRS1 and CES-D/RRS was significantly higher than the correlation between PBRS2 and CES-D/RRS (*z* = 5.986, 3.526, all *p* < 0.01).

**Continued Table 3:** Correlation matrix of modified PBRS and NBRS, original PBRS and NBRS, and self-report scales (n = 1025)

	oPBRS	oNBRS	oNBRS1	oNBRS2
oNBRS	0.303^**^	-		
oNBRS1	0.334^**^	0.963^**^	-	
oNBRS2	0.153^**^	0.813^**^	0.627^**^	-
CESD	0.197^**^	0.459^**^	0.439^**^	0.382^**^
MCQ2	0.371^**^	0.059	0.088^**^	-0.020
MCQ4	0.312^**^	0.576^**^	0.578^**^	0.419^**^
RRS	0.482^**^	0.503^**^	0.502^**^	0.372^**^
PSWQ	0.303^**^	0.438^**^	0.447^**^	0.301^**^
*z*-test	6.488^**^	2.484^*^	2.108^*^	2.492^*^

Note: oPBRS = original Positive Beliefs about Rumination Scale, oNBRS = original Negative Beliefs about Rumination Scale, oNBRS1 = original subscale 1 of NBRS (uncontrollability and harmfulness), oNBRS2 = original subscale 2 of NBRS (interpersonal and social consequences). ^**^ significant at the 0.01 level. ^*^ significant at the 0.05 level.

For the modified NBRS (mNBRS) scales, the concurrent validity was provided by positive and moderate correlations between these scales and MCQ4. At the same time, there were none to minor significant correlations between all the modified NBRS scales and MCQ2. Support for the convergent validity of the mNBRS scales was obtained from significant positive correlations with the CES-D and RRS. The divergent validity of NBRS1 was also affirmed by a significantly higher correlation with the RRS than with the PSWQ, which specifically measures proneness to anxious worry. But for another two mNBRS subscales (NBRS2 and NBRS3), this significant strength of association did not be found (*t*-test in Table 3). To be able to compare these results to those of previous research, the original one-factor model of the PBRS and the two-factor model of the NBRS were also tested and reported.

### Reliability

Internal consistency was evaluated using Cronbach’s *α*, which was regarded as unacceptable when it was below 0.6 [60]. The alpha reliabilities of both modified PBRS factors were high (Understanding feelings and causes: *α* = 0.846 for PBRS1; Problem discovering and preventing: *α* = 0.865 for PBRS2). For the modified NBRS subscales, the alpha indexes were acceptable (Consequences of rumination: *α* = 0.779 for NBRS1; Uncontrollability of rumination: *α* = 0.632 for NBRS2; Self-appraisal with rumination: *α* = 0.708 for NBRS3).

The test–retest reliability was tested using the 3-week ICC. Respectively, the reliability coefficients for the modified subscales of PBRS (PBRS1 and PBRS2) and the modified subscales of NBRS (NBRS1, NBRS2, and NBRS3) were 0.803, 0.842, 0.773, 0.806, and 0.681, respectively. According to the recommendation, these results indicated substantial to almost perfect stability [59].

For the original PBRS and NBRS, their internal consistency index was acceptable (0.905 for oPBRS, 0.787 for oNBRS1, 0.758 for oNBRS2), as well as their ICC (0.879 for oPBRS, 0.844 for oNBRS1, 0.767 for oNBRS2).

### Gender differences

Independent samples *t*-tests between genders were performed. For the modified PBRS and NBRS subscales, males obtained significantly higher scores than females on the two factors of the modified PBRS, while there were no significant differences between males and females for the modified NBRS factors. This gender difference was also found in the original PBRS but not in the original NBRS.

## Discussion

The current study aimed to establish an initial Chinese version of the PBRS and the NBRS. The original English versions were translated and back-translated for appropriate adjustment. For both the new modified models and the original models of these two scales, EFA, CFA, validity (concurrent, convergent, and divergent), and reliability (internal consistency and test–retest) were explored in sequence. Similar results for the original models indicated the reliability of the data, which further demonstrated the acceptable psychometric properties of the new modified models of the Chinese versions of the PBRS and the NBRS.

First, the EFA results indicated that the data fit best with the two-factor structure of the Chinese PBRS and the three-factor structure of the Chinese NBRS. Item 4 of the PBRS and item 7 of the NBRS were omitted because of poor factor loading.

For the PBRS, the two modified factors were called “Understanding feelings and causes” (PBRS1, Understanding) and “Discovering and preventing problems” (PBRS2, Problem-solving). Obviously, this structure is more elaborate compared to the positive beliefs subscale of the MCQ, which only focuses on the function of problem-solving without mentioning beliefs about understanding things [61]. Moreover, compared to the original one-factor PBRS, this two-factor structure is more aligned with the classical definition of rumination, which also contained the causal and consequential aspects [47]. Previous research has provided support for this modified structure. In a cross-sectional study of five samples from two cultures (total *N* = 1414), the researchers regarded rumination as an adaptive cognitive process consisting of two analytical factors: causal analysis (CA) and problem-solving analysis (PSA) [62]. Further research found that depressed individuals might engage in more CA than non-depressed individuals [63], which is in line with our findings that PBRS1 is significantly more correlated to negative beliefs, rumination, depressive symptoms, and subscales of the NBRS than those between PBRS2 and these negative scales. Accordingly, preserving this two-factor model might contribute to the development of rumination and depression in a different way.

For the NBRS, the three modified factors were respectively termed “Consequences of rumination” (NBRS1, Consequences), “Uncontrollability of rumination” (NBRS2, Uncontrollability), and “Self-appraisal influenced by rumination” (NBRS3, Self-appraisal). Although the new modified NBRS seems quite different from the original, in fact, it can be regarded as reallocating the original factors. It separated the original first subscale of the NBRS into two factors: harmfulness (NBRS1) and uncontrollability (NBRS2); transferred the items relating to interpersonal consequences from the original second subscale of the NBRS into the harmfulness factor (NBRS1); and left the items relating to self-appraisal to create the third factor (NBRS3). These modifications make the structure of NBRS more specific when considering individuals’ metacognitive beliefs about uncontrollability and about their current cognitive function. Evidence that depressive rumination is associated with deficits in cognitive function has been found [64]. Therefore, as the MCQ includes a factor to measure individuals’ cognitive confidence, this modification of the original structure of the NBRS suggests that beliefs about cognitive control could be an important component of individuals’ metacognitive beliefs. Moreover, the items of NBRS3 that describe the influence of rumination on people’s self-appraisal were coincidentally consistent with several items (e.g., items 23, 2, and 8) of the Automatic Thoughts Questionnaire (ATQ) [65]. As prior studies have shown, these automatic thoughts, which reflect negative content about the self, are considered a relatively stable vulnerability associated with depression but not anxiety [66, 67]. Therefore, this kind of rumination could be a specific factor in depressive individuals’ metacognition.

Generally, items in the NBRS were in the same factors as the original model, except that item 13 (“Ruminating can make me harm myself”) was classified as a self-appraisal factor rather than a consequence factor, which was inconsistent with previous studies [33]. The item “harm myself” could be understood as self-injury behavior. Non-suicidal self-injury includes mild to moderate damage to the body and beliefs about being unacceptable to the community, and it seems that how others evaluate this behavior is more important than actual injury [68]. Studies have shown that individuals tend to hide their self-injury behaviors from others and regard these behaviors with a sense of self-depreciation, shame, and guilt [69]. Their self-injury behavior can be predicted by rejection sensitivity [70], the tendency of anxious expectation, and overreaction to interpersonal rejection cues [71].

Moreover, other items of the NBRS including item 8 (“Ruminating will turn me into a failure”) and item 12 (“Only weak people ruminate”) were considered as self-appraisal factor could also be explained by cultural specificity. A recent study investigated the attitude of Chinese people toward depression using the Depressive Stigma Scale (DSS); results showed that the highest scored item in the DSS was “People with depression could snap out of it if they wanted” [72], which suggested that many Chinese tend to believe that individuals are capable of getting themselves out of mental disorders. Individuals may feel frustrated and disappointed with themselves if they have failed to control or are unable to overcome their own “troubles” [73] and consider themselves “a failure”(item 8) or “weak people” (item 12).

Another explanation could be that individuals with mental disorders such as depressive disorder, anxiety disorder [74], and schizophrenia [75], are likely to be stigmatized in many societies [76], especially in China [77]. This stigma could be affected by family relations and the social climate. Chinese culture places great emphasis on the concept of collectivism [78]. In a collectivist society, people are more likely to approve of public stigma toward mental disorders. Though there are growing media guidelines about mental health problems in China, many reports are biased and reinforce the notion that people with mental disorders are dangerous, unpredictable, incapable, and unreliable [79]. These public notions may lead to high perceived stigma, which may result in people being more sensitive to the label of mental disorder that might damage their own and family reputations [80]. Taking our participants’ cultural backgrounds into consideration, therefore, it is understandable that these items are treated as being more associated with the social and self-appraisal aspects than the actual physical consequences.

The CFA showed a good fit of the modified two-factor model of the PBRS and the three-factor model of the NBRS. For these two scales, two items (5 and 9) of the PBRS and two items (4 and 5) of the NBRS had residual correlations, indicating they might have similar expressions and/or a strong association of expression in Chinese. Concerning the content of these items, item 5 in the PBRS (“prevent future mistakes”) could be regarded as the effect of item 9 (“work out how things could have been done better”). Item 4 in the NBRS (“everyone would desert me”) expressed a quite parallel meaning (e.g., interpersonal rejection) with item 5 in the NBRS (“people will reject me”). After allowing these items to have residual correlations, the fit of the models was significantly improved. Based on the EFA and CFA results, considering Chinese culture, the modified two-factor model of the PBRS and the modified three-factor model of the NBRS seemed to be more suitable for further research.

Both the PBRS and NBRS achieved good indices of validity. There were positive and significant associations between all subscales of the modified PBRS and the MCQ2 used to assess individuals’ positive beliefs about worry and to test the construct validity of the PBRS. In accordance with previous reports [1, 54], these meaningful associations also appeared between PBRS scores and rumination, thus supporting the concurrent validity of the PBRS. Furthermore, the significant differences in the correlations between the two modified PBRS subscales and other self-reported measurements may suggest a potentially complicated mechanism in the effects of positive beliefs about rumination on rumination and depression. The discriminant validity of the modified PBRS was supported by the significant difference in the association between PBRS and rumination and between PBRS and worry.

Regarding the modified NBRS, there were positive and significant associations between all subscales of the modified NBRS and MCQ4 used to measure individuals’ negative beliefs about worry and to test the construct validity of the NBRS. The concurrent criterion validity was supported by the significant correlation between all the NBRS scales and self-reported depressive symptoms and rumination. It is of note that the associations between the three NBRS scales and rumination and worry did not differ significantly, except for NBRS1 (Consequences of rumination). Regarding the NBRS2, this result may be because negative beliefs about the uncontrollability of thoughts are related to repetitive negative thinking in general [54]. Regarding the NBRS3, this result may be due to automatic thoughts, particularly regarding one’s weakness and failure, related to both rumination and worry. Considering the previous studies that reported that the association between automatic thoughts and rumination is closer than the association between automatic thoughts and worry [66, 67], we believe that the addition of some distinguishing items about self-appraisal might be helpful in improving the divergent validity of the NBRS in future research.

The results of this current study indicate that both the PBRS and the NBRS are reliable tests to measure positive and negative beliefs about rumination. The alpha coefficients were all above 0.63, which was good for the two PBRS factors (0.846–0.865) and acceptable for the three NBRS factors (0.632–0.779). According to the recommendation of Shout [59], the retest results after three weeks led to good retest reliability of the PBRS scales (0.803 < ICC < 0.842) and moderate to good retest reliability of the NBRS scales (0.681 < ICC < 0.806).

The result that males elicited higher PBRS scores than females was not surprising, as in previous studies reports of gender differences in the PBRS score in non-depressed samples were inconsistent. Papageorgiou and Wells reported that women showed a slightly higher but not significant PBRS score than men [1], while [83]Williams and Moulds reported a slightly higher score for males than females in the never-depressed sample [81]. This may partly be due to men’s stronger sense of control over important events in their lives compared to women [82] and less effort when using cognitive emotional regulation [83]. Men may be more likely than women to engage in problem solving in attempting to control or change the situations they believe are driving their emotions [84].

As previous studies have suggested, metacognitive beliefs and rumination explained significant variance of depression in their theorized order: positive beliefs about rumination predicted rumination which again predicted negative beliefs about rumination which again predicted depressive symptoms in the population [85]. [89]Additionally, both positive and negative beliefs showed moderate associations with rumination, but positive beliefs showed low associations with depression whereas negative beliefs showed moderate associations with depression [7]. Compared to non-depressed students, the effect of negative beliefs on depression in clinically depressed individuals increased [86]. According to these results, the association between depression and negative beliefs might be stronger than depression and positive beliefs, which indicate that negative beliefs about rumination might potentially cause individuals’ depressive symptoms to deteriorate.

MCT has been proven useful in decreasing rumination and depression [22]. Interventions that stimulate a decrease in rumination and depression might contain potential metacognitive mechanisms, such as mindfulness practices that may help in promoting sustained attention and developing cognitive control [87], as well as reducing stigma [88] and negative metacognitions [89]. Since more elaborate structures of metacognition have been found, corresponding specific techniques to decrease negative metacognitions from different perspectives may facilitate treatments that produce promising and efficient results [90, 91]. [97, 98]

As the PBRS and NBRS have been used in combination in previous studies, it is worth exploring whether these two scales could be merged into one. Our results showed that the significant correlation among subscales of the PBRS and the NBRS is promising evidence that supports their combination. Except for the fact that the correlation between PBRS2 and NBRS2 was below 0.1, other correlative indexes among these factors were close to the correlation between subscales of the MCQ, which ranged from 0.21 to 0.65 [61, 92]. These widely varied correlations might be partly due to the relatively few items for each factor.

This study has several limitations. Our samples consisted of Mandarin-speaking undergraduates with similar ages and education levels who may lack diversity and cannot be representative of individuals within the community or of patients with affective disorders. Meanwhile, we didn’t investigate participants’ financial backgrounds or the environment in which they raised (rural or urban), which may have impacted the attitudes and behavior toward mental illness [93]. Therefore, certain problems with the generalizability of the research results may occur. Future studies should examine the psychometric properties of the PBRS and NBRS in different populations. Another limitation of this study is its cross-sectional design, which makes it difficult to reveal the causality of the relationship between beliefs about rumination, depressive rumination, and depression. Therefore, longitudinal studies are needed to address these questions. Moreover, considering the S-REF model and the special role of negative metacognitive beliefs, larger and controlled interventions must be explored in the future.

## Conclusion

The current study provided evidence that the Chinese versions of the PBRS and the NBRS are generally reliable and valid, while the two-factor structural model of the PBRS and three-factor model of the NBRS are more suitable for Chinese college students. Considering the unneglectable impact of culture on individuals’ metacognitive beliefs, these new models of PBRS and NBRS are of value to be further explored in Chinese population .

## Data Availability

The datasets used and analyzed during the current study available from the corresponding author (deng0087@126.com) on reasonable request.
